# The Roles of Two Type VI Secretion Systems in *Cronobacter sakazakii* ATCC 12868

**DOI:** 10.3389/fmicb.2018.02499

**Published:** 2018-10-22

**Authors:** Min Wang, Hengchun Cao, Qian Wang, Tingting Xu, Xi Guo, Bin Liu

**Affiliations:** ^1^Key Laboratory of Molecular Microbiology and Technology, Ministry of Education, Tianjin Economic-Technological Development Area, Nankai University, Tianjin, China; ^2^Tianjin Key Laboratory of Microbial Functional Genomics, Tianjin Economic-Technological Development Area, Nankai University, Tianjin, China; ^3^TEDA Institute of Biological Sciences and Biotechnology, Tianjin Economic-Technological Development Area, Nankai University, Tianjin, China

**Keywords:** *Cronobacter sakazakii*, type VI secretion system (T6SS), virulence, antibacterial activity, isogenic mutants

## Abstract

The type VI secretion system (T6SS), which has been found in 25% of gram-negative bacteria, is a crucial virulence factor in several pathogens. Although T6SS gene loci have been discovered in *Cronobacter* species, one of the major opportunistic foodborne pathogens, its function has not been elucidated. In this study, the roles of two phylogenetically distinct T6SS gene clusters in *Cronobacter sakazakii* ATCC12868 were investigated. Analysis of 138 genome sequences of *C. sakazakii* strains, we found that one T6SS gene cluster (T6SS-1) was ubiquitous in all examined strains, whereas another (T6SS-2) was absent or degenerated in a large proportion of the strains (*n* = 97). In addition, we confirmed the T6SS-1 antibacterial function through an in-frame deletion in the *vasK* and *hcp* genes. Compared with the wild-type strain, the T6SS-2-deficient mutant presented a much stronger colonization of organs when infecting neonatal rats. Thus, we proposed that T6SS-2 plays a role in pathogenic processes. This is the first study to investigate the functions of T6SS in *C. sakazakii*, and the results will extend our understanding of the pathogenic and phylogenetic characteristics of *C. sakazakii*.

## Introduction

*Cronobacter* spp. is an emerging opportunistic food-borne gram-negative pathogen known to cause severe clinical infections in neonates, including necrotizing enterocolitis (NEC), sepsis, and meningitis (Biering et al., 1989; Gallagher and Ball, 1991; Caubilla-Barron et al., 2007). Infections by *Cronobacter sakazakii* have been reported only in infants, the elderly and immunocompromised adults (Healy et al., 2010; Hunter and Bean, 2013). Neonates with poor immunity or low-birth weight are the most susceptible population, often acquiring the infection by consuming contaminated powdered infant formula (Muytjens et al., 1983; Tall et al., 2014). *Cronobacter* spp. have caused several outbreaks of neonatal meningitis and necrotizing enterocolitis, resulting in a high mortality rate (approximately 33–80%) (Lai, 2001; Healy et al., 2010) and serious sequelae such as brain abscesses and impaired sight and hearing (Kleiman et al., 1981; Muytjens et al., 1983).

Type VI secretion system (T6SS) has been found in over 25% of sequenced gram-negative bacterial strains (Bingle et al., 2008). Structurally, the organelle is analogous to a contractile phage tail, which is comprised of 12 to more than 20 proteins. T6SS core components consist of 13 conserved proteins. Among them, VasK is a membrane-associated protein with ATPase activity, and is essential for a functional T6SS apparatus (Ma et al., 2009). Hcp is one of the components of the T6SS phage tail, which can also be delivered as an effector (Bingle et al., 2008; Russell et al., 2014).

Type VI secretion system (T6SS) is a versatile protein secretion apparatus that can directly deliver toxins into eukaryotic cells as well as other bacteria. Their functions are associated with virulence, host immunity resistance and interbacterial interaction. The T6SS of *Pseudomonas aeruginosa* can secrete three kinds of effectors (Tse1-3), which can destroy peptidoglycans, cell membranes, and cytoplasmic components in infected cells (Russell et al., 2011; Russell et al., 2013). T6SS genes are required for virulence of *Vibrio cholerae* toward *Dictyostelium amoebae* and macrophages (Pukatzki et al., 2006). *Yersinia pseudotuberculosis* resists host immunity through the transport of Zn^2+^ in a T6SS-dependent mechanism (Wang et al., 2015). In addition, T6SS demonstrates antivirulent characteristics in some species (Chow and Mazmanian, 2010; Bendor et al., 2015). In *Bordetella bronchiseptica*, a T6SS-deficient mutant exhibits a hypervirulent phenotype when infecting immunodeficient mice (Bendor et al., 2015).

Through whole-genome analysis, several putative T6SS loci have been discovered in *Cronobacter* spp. (Joseph et al., 2012). However, their functions are not yet understood. By genomic analysis of 138 *C. sakazakii* strains, two integrated T6SS loci were found namely T6SS-1 and T6SS-2. T6SS-1 was ubiquitous among our examined strains, whereas the T6SS-2 gene cluster is absent or degenerated in approximately 70% (97/138) of the strains. In addition, approximately 80% (23/29) of clinical strains are T6SS-2-negative. Therefore, we sought to answer the question of whether T6SS-1 plays an essential role during strains’ growth and infection, whereas T6SS-2 is redundant in their routine niches. To answer this question, T6SS-deficient strains were constructed by deletion of *vasK* and *hcp* genes. The properties of interbacterial competition, human intestinal epithelial cell invasion, human macrophage intracellular survival, and neonatal rat infection between wild-type and mutant strains were evaluated. The findings of this study would shed more light on the role of T6SS in the pathogenesis of *C. sakazakii* and enable future development of therapeutic strategies to combat *C. sakazakii* infections.

## Materials and Methods

### Bacterial Strains and Cell Lines

The bacterial strains and plasmids used in this study are listed in Supplementary Table S1. *C. sakazakii* (ATCC12868), Caco-2 cells and U937 cells were obtained from ATCC. Caco-2 cells and U937 cells were cultured in minimal essential medium (MEM) and RPMI 1640 (Gibco), respectively, and supplemented with 10% fetal bovine serum (Life Technologies) in a 5% CO2 atmosphere at 37°C. For competition experiments, the strains were grown in Luria Bertani (LB) broth at 37°C with shaking, and when required, antibiotics were added at the following concentrations: ampicillin (100 μg/mL), chloramphenicol (20 μg/mL), and streptomycin (100 μg/mL) (Sigma).

### Distribution of the Two T6SS Gene Loci in *C. sakazakii* Strains

A total of 138 genome sequences of *C. sakazakii* were examined. All genomes were compared against the genome of ATCC12868, which contains the two integral T6SS loci. The presence of the two T6SS loci in each individual genome was assessed using the Artemis Comparison Tool (ACT) (Carver et al., 2005).

### T6SS Gene Mutation and Complementation

Mutant strains containing deletions of *vasK1*, *hcp1*, *vasK2*, and *hcp2* were constructed using the λ-red recombinase system (Datsenko and Wanner, 2000). Briefly, PCR primers (Supplementary Table S2) contained the sequences corresponding to the end of the desired deletion, whereas the 20 nucleotides at the 3′ end contained the sequence of the chloramphenicol (cam) drug resistance cassette from the plasmid pKD3. Plasmid pKD46 was utilized to synthesize recombinase. Complementation experiments were performed by cloning the respective genes into a pTrc99A vector with IPTG-induced expression on LB-agar plates containing 1m M IPTG.

### Growth Curve

Strains were incubated overnight and then transferred into 100 ml of fresh LB at a ratio of 1:100. The strains were then grown at 37°C with shaking at 175 rpm/min. OD600 was measured every 30 min for each strain.

### Bacterial Competition Assay

Streptomycin-resistant derivatives of prey strains were generated by spontaneous mutation as previously described (Johnson et al., 2005). Competition experiments were then performed as previously described (MacIntyre et al., 2010). In brief, streptomycin-sensitive predator and streptomycin-resistant prey bacteria were mixed at a 1:1 or 10:1 ratio. Approximately 10^8^ bacteria were then spotted on dry LB-agar plates and incubated at 37°C for 1–4 h. The bacteria were harvested, diluted, and plated on LB plates containing 100 μg/ml of streptomycin. Each experiment was performed in duplicate and repeated thrice.

### Caco-2 Cell Invasion Assay

We used a gentamicin protection assay to determine the number of intracellular bacteria, which was performed as described previously (Kim and Loessner, 2008) with some modifications. Briefly, Caco-2 cells were seeded in 6-well plates. After 24 h, the monolayer of cells was infected with mid-exponential phase bacteria at a multiplicity of infection (MOI) of 10 for 90 min in an incubator at 37°C with 5% CO_2_. The infected cells were washed three times with sterile phosphate-buffered saline (PBS), and then fresh medium containing gentamicin (100 μg/ml) was added. The plate was incubated for 1 h at 37°C with 5% CO_2_ and then washed three times with PBS. The infected cells were lysed with 0.1% Triton X-100 for 10 min. The bacteria were then collected and plated onto LB agar using 10-fold serial dilutions. Each experiment was performed in duplicate and repeated thrice.

### Human Macrophage Invasion and Intracellular Survival Assay

The gentamicin protection assay and intracellular survival assay were performed as previously described (Townsend et al., 2007). In brief, U937 cells were seeded in 24-well plates with phorbol 12-myristate 13-acetate (PMA). After 48 h, cells were gently washed with RPMI to remove residual PMA. Monolayer cells were infected with mid-exponential phase bacteria at a MOI of 10 for 1 h at 37°C with 5% CO_2_. The infected cells were washed two times with PBS. Fresh medium containing gentamicin (100 μg/ml) was added, and the plate was incubated for 1 h at 37°C with 5% CO_2_, and then washed three times with PBS. Fresh medium containing gentamicin (10 μg/ml) was added, with the cells being incubated continually at 37°C with 5% CO_2_. The infected cells were then lysed with 0.1% TritonX-100 at time points 0, 12, 24, 36, and 48 h. The bacteria were collected and plated onto LB agar using 10-fold serial dilutions.

### Neonatal Rat Experiments

All animal experiments were performed according to the standards of the Guide for the Care and Use of Laboratory Animals(Council, 2011). Experimental protocols were approved by the Institutional Animal Care Committee at Nankai University. Animal experiments were conducted as previously described (Mittal et al., 2009). Briefly, 4-day-old Sprague–Dawley rat pups from one mother were randomly divided into several groups and infected orally with 10^4^ CFU of wild-type *C. sakazakii*, T6SS-deficient strains, and complemental strains in 30 μl of PBS. The control group was fed with PBS. The rats were euthanized 48 h after infection. Brain, liver, and spleen were aseptically removed and homogenized in sterile PBS. Bacterial counts in the tissue homogenates were determined by plating 10-fold serial dilutions on chloramphenicol-, ampicillin-, or streptomycin-LB agar plates.

### Ethics Statement

All animal experiments were carried out according to the standards set forth in the Guide for the Care and Use of Laboratory Animals published by the Institute of Laboratory Animal Resources of the National Research Council (Untied States). The experimental protocols were approved by the Institutional Animal Care Committee at Nankai University. We have made efforts to minimize animal suffering and reduce the number of animals used.

## Results

### Distribution and Genetic Structure of T6SS Gene Loci

**FIGURE 1:**
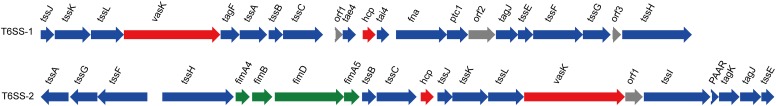
Schematic representation of the two T6SSs gene loci in *Cronobacter sakazakii*. Core components of the T6SSs are shown in blue. Uncharacterized genes are shown in gray. In the T6SS-2 cluster, fimA4, fimB, fimD, and fimA5 are pilus-associated genes. The mutant genes in the experiment are shown in red.

The annotations for available T6SS clusters and their components were based on the SecReT6 database^1^, combined with manual checking. In the ATCC 12868 strain, two intact T6SS loci were found and named T6SS-1 and T6SS-2 (Supplementary Table S3). T6SS-1 contained 21 contiguous genes, including 18 conserved core components and 3 accessory genes (Figure 1). The *ptc1* has been shown to play a regulatory function in *P. aeruginosa* (Mougous et al., 2007). The putative peptidoglycan amidase toxin-antitoxin combination and phospholipase genes are also located inside the cluster, and are antibacterial effectors in *E. cloacae*, *S. typhimurium*, and *P. aeruginosa* (Russell et al., 2013; Zhang et al., 2013). Therefore, this suggests the T6SS-1 may have an antibacterial function. A total of 15 core component genes were found in T6SS-2, with no regulatory and effector genes (Figure 1). Therefore, the function of T6SS-2 is far from clear.

**FIGURE 2:**
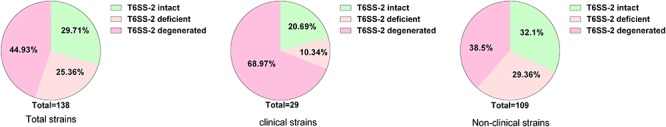
The distribution of T6SS-2 in 138 *C. sakazakii* strains, including clinical and non-clinical strains. The genome of each strain was compared to that of ATCC12868. Strains that lack the 13 essential core components of T6SS-2 are classified into the deficient group, while strains with the loss of several of *vasK, tssH, hcp* or other important genes partially, or entirely are defined as degenerated.

A total of 138 *C. sakazakii* genome sequences were used to investigate the distribution of the two T6SS clusters. A total of 96 *C. sakazakii* genomes were sequenced by our lab, and the remaining 42 were obtained from NCBI database. The results showed that an intact T6SS-1 gene locus was present in all 138 strains, whereas an intact T6SS-2 was only found in 41 (29.7%) strains. Approximately 25% (35/138) of the strains had lost their entire T6SS-2 locus, and 44.9% (62/138) of the strains were T6SS-2-degenerated, with the *vasK, hcp*, and/or *tssH* genes being absent or present as a pseudogene. In addition, approximately 80% (23/29) of the clinical strains contained a deficient or degenerated T6SS-2 cluster (Figure 2). All strains were responsible for a fatal clinical disease, such as 701, 767, 695, and NM1240, contained truncated *tssH* and *vasK* genes, which implied that their T6SS-2 was non-functional. *tssH* is predicted to be a type VI secretion system ATPase which plays an important role in sheath recycling (Brodmann et al., 2017). These data suggest that loss of T6SS may be beneficial to *C. sakazakii* infection in neonates.

### Wild-Type and Two T6SS-Deficient Strains Exhibit Similar Growth Rates

In other pathogens, it was shown that deletion of the *vasK* and *hcp* genes can inactivate T6SS (Mougous et al., 2006; Pukatzki et al., 2006). Therefore, T6SS-1- and T6SS-2-deficient strains were created by deleting of *vasK* or *hcp* gene. The deletion of T6SS genes had no effect on the bacteria’s growth rate (Supplementary Figure S1), indicating that any differences between the wild-type and the two T6SS-deficient strains were not a result of differences in the growth rate.

### ATCC12868 Kills Other Gram-Negative Bacteria in a T6SS-1-Dependent Manner

**FIGURE 3:**
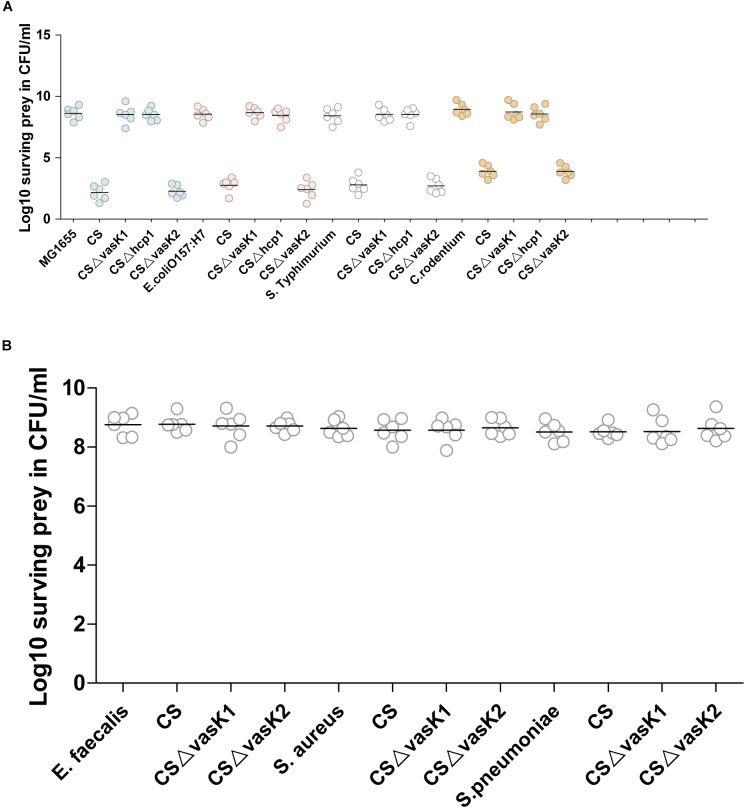
*C. sakazakii* targets gram-negative species in a T6SS-1-dependent manner. Survival of streptomycin-resistant prey is shown. **(A)** Streptomycin-sensitive predators and streptomycin-resistant gram-negative preys were mixed at a 10:1 ratio and incubated for 4 h. The surviving preys were counted by plating on agar containing 100 μg/ml streptomycin and is presented as Log10 CFU. **(B)** The Gram-positive preys were counted using the same method as **(A)**. The data represent three independent experiments.

**FIGURE 4:**
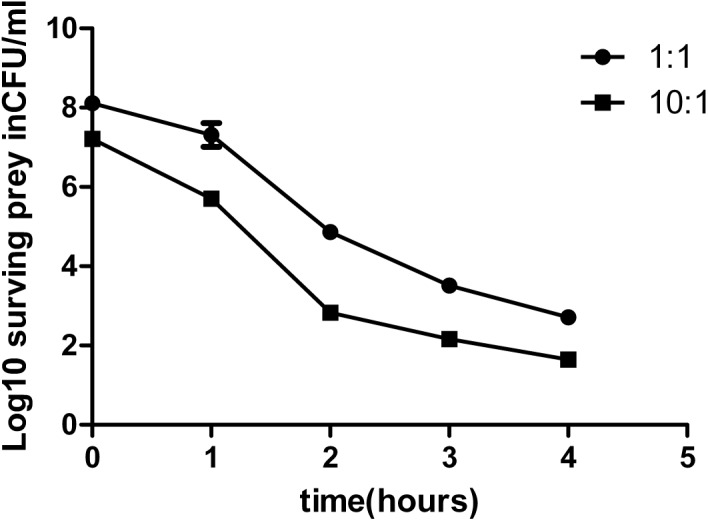
Survival curve of *E. coli* K12. Predators and prey were mixed at a ratio of 1:1 or 10:1. Survival of prey was measured at several time points. The data represent the mean (±SD) of experimental duplicates.

We first performed an experiment to determine whether the T6SS of *C. sakazakii* had antibacterial functions similar to those of T6SS in other pathogens such as *V. cholerae* and *P. aeruginosa* (Hood et al., 2010; MacIntyre et al., 2010). *Escherichia coli* K-12, *E. coli* O157:H7 (EHEC), *Salmonella typhimurium* and *Citrobacter rodentium* were selected as the gram-negative prey strains, and *Enterobacter faecalis*, *Staphylococcus aureus*, and *Streptococcus pneumoniae* were selected as the gram-positive prey strains. Wild-type, T6SS-1-deficient, and T6SS-2-deficient strains were co-cultured with these prey strains for 1–4 h. The wild-type strain and T6SS-2-deficient strain were highly virulent toward the Gram-negative bacteria. However, this virulence was abrogated when either the *vasK1* or *hcp1* gene was deleted (Figure 3A). Unsurprisingly, gram-positive bacteria were resistant to killing by *C. sakazakii* (Figure 3B). To confirm T6SS-1 mediated virulence toward gram-negative strains is in a T6SS-dependent manner, *E. coli* O157:H7 was selected as experimental prey for future experiments. The result showed survival of *E. coli* was restored in the complemented strains H6561 and H6563 (Supplementary Figure S2). The survival curves showed that *C. sakazakii* had the highest killing efficiency during the second hour of infection (Figure 4). These results suggested that T6SS-1 of *C. sakazakii* had antibacterial functions similar to its counterparts in *V. cholerae* and *P. aeruginosa*, whereas T6SS-2 did not exhibit antibacterial function.

### Wild-Type and T6SS-Deficient Strains Exhibit Similar Caco-2 Invasive Efficiencies

**FIGURE 5:**
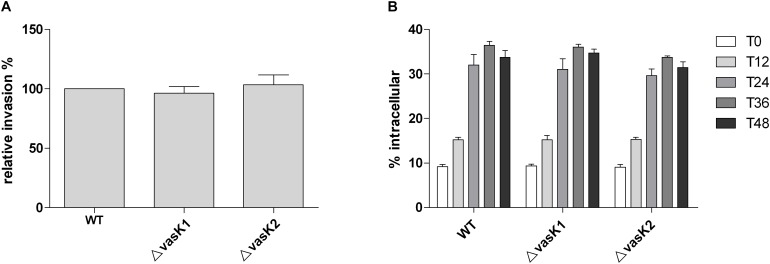
Contribution of the two T6SSs to *C. sakazakii* human intestinal epithelial cell (Caco-2) invasion and human macrophage U937 intracellular survival. **(A)** Caco-2 cells were infected at a MOI of 10 for 90 min. Bacteria were recovered after a 1 h gentamicin protection assay. The results are presented as relative percentages. The error bars indicate standard deviations for the means of three separate experiments performed in triplicate. **(B)** U937 cells were infected at a MOI of 10 for 60 min. The intracellular bacterial numbers are described as T0. After a 1 h gentamicin protection assay, intracellular bacteria were recovered at time points of 12, 24, 36, and 48 h. The results are presented as the percent intracellular of the inoculum. Data are the means ± standard error of two independent experiments performed in triplicate.

In some pathogens, T6SS is involved in host cell invasion (Zhou et al., 2012). Additionally, it has been previously shown that the presence of traversing intestinal epithelial cells is required for *C. sakazakii* to cause sepsis and meningitis. Therefore, we assessed whether *C. sakazakii* invaded Caco-2 cells in a T6SS-dependent manner. After 90 min of incubation of Caco-2 monolayer cells with the wild-type, Δ*vasK*1 andΔ*vasK*2 strains, respectively, and 1 h of gentamicin treatment, intracellular survival was assessed. No significant difference in invasive efficiency between the wide-type and the two T6SS deletion mutant strains was observed (Figure 5A). These results demonstrate that neither T6SS-1 nor T6SS-2 were involved in the invasion of intestinal epithelial cells.

### Wild-Type and T6SS-Deficient Strains Have the Same Competence in Macrophage Invasion and Intracellular Survival

In pathogens such as *V. cholerae*, *P. aeruginosa*, and *S. enterica*, T6SS is involved in the invasion and intracellular survival of pathogens within macrophages (Pukatzki et al., 2007; Blondel et al., 2013). *C. sakazakii* can survive and multiply in macrophages for a relatively long time (Townsend et al., 2007). Therefore, we tested whether the two T6SSs played a role in the invasion and survival of *C. sakazakii* in macrophages. Bacterial were recycled at 1, 12, 24, 36, and 48 h post-infection. Eventually, the two T6SS-deficient strains (Δ*vasK*1 and Δ*vasK*2) exhibited similar invasion abilities (T0) and intracellular reproduction tendencies (T12, T24, T36, and T48) (Figure 5B). These results suggested that neither T6SS-1 nor T6SS-2 affected *C. sakazakii* survival in human macrophages.

### T6SS-2-Deficient Strain Exhibits a Hypervirulent Phenotype in Neonatal Rats

**FIGURE 6:**
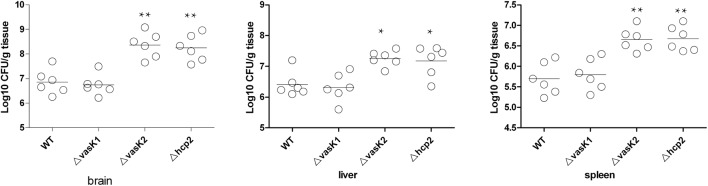
Bacterial colonization in tissues of neonatal rats infected with wild type or the two T6SS gene mutants, respectively. Groups of 4 days old neonatal rats (*n* = 6) were orally infected with 10^4^ CFU/30 μl strains. Brains, livers and spleens were harvested at 48 h post-infection. Equal weights of tissues were homogenized and plated on LB agar containing 20 μg/ml chloramphenicol or 100 μg/ml streptomycin. The number of bacteria were counted and expressed as Log10 CFU/g tissue ± SD. All *P*-values were determined using Mann-Whitney test. ^∗^*P* ≤ 0.05; ^∗∗^*P* ≤ 0.01.

The ATCC12868 strain has been documented to cause meningitis (Townsend et al., 2007). We used a neonatal rat model to investigate the virulence of wild-type and T6SS-deficient strains in animals. The 4-day-old rats were orally fed with 10^4^ CFU/30 μl of either wild-type or T6SS-deficient strains. Bacteria were recovered from the brain, liver, and spleen at 48 h post-infection. Our results showed that similar numbers of T6SS-1-deficient and wild-type bacteria were recovered from different organs. However, T6SS-2-deficient bacteria were collected from brains at about a 10-fold higher number compared to that of wild-type. The numbers were also higher in the liver and spleen but to a slightly lesser extent than in the brain (Figure 6). Complemental strains also showed a significant difference from the deficient strains (Supplementary Figure S3). These results suggest that T6SS-2 might limit the ability of *C. sakazakii* to invade or grow in host organs.

## Discussion

The T6SS-1 cluster is ubiquitous among *C. sakazakii* strains, and its GC content (59.64%) is similar to that of the whole genome (57.02%), which suggests that the gene cluster is part of the inherent genetic material of the species. The antibacterial function of T6SS-1 may be important for the species to gain survival advantages in both environmental and host niches, as it is in several other pathogens such as *V. cholerae, P. aeruginosa*, and *Serratia marcescens* (MacIntyre et al., 2010; Russell et al., 2011; Alcoforado Diniz and Coulthurst, 2015). In addition, we propose that the antibacterial function has a more profound significance for *C. sakazakii* during host infection, especially as a cause of NEC in newborns, as the most vulnerable targets of *C. sakazakii* are neonatal infants that have low complexity and diversity in their fluid gut microbiota (Grishin et al., 2013). We hypothesize that *Cronobacter* kills gram-negative species after infection, further reducing the complexity and diversity of an already frail gut microbiota. The relation between NEC and gut microbiota is still unclear (Grishin et al., 2013), and further research is needed to determine whether the antibacterial function of T6SS-1 enhances the ability of *C. sakazakii* to cause neonatal NEC.

In this study, we found that the deletion of T6SS-2-associated genes is beneficial to *C. sakazakii* during its infection of neonatal rats, implying that the T6SS-2 has an anti-virulent function. Similar anti-virulence functions have been found in other pathogens (Parsons and Heffron, 2005; Kinkel and McIver, 2008; Chow and Mazmanian, 2010; Li et al., 2014; Bendor et al., 2015). For example, T6SS is required for *B. bronchiseptica* to infect wild-type mice; however, a T6SS-deficient mutant exhibits a hypervirulent phenotype when infecting immunodeficient mice (Bendor et al., 2015). In *S. typhimurium*, SciS (*vasK* homolog) reduces intracellular bacterial numbers at later stages of infection and attenuates virulence to achieve a balance within the host environment (Parsons and Heffron, 2005). The T6SS-2 cluster has a much lower GC content (51.02%) than the whole genome and contains pseudogenes in a large proportion of *C. sakazakii* strains (97/138), which suggests that the species is losing this gene cluster under an unknown selective pressure.

This work is the first to describe the function of T6SS in *Cronobacter spp*. We found that the two T6SS were different in both function and distribution among *C. sakazakii* strains. To our knowledge, this is the first report of T6SS-1 especially contributing to interbacterial competition, which might be crucial for *C. sakazakii* to compete with other species in their various niches. The T6SS-2 cluster might be important for *C. sakazakii* during host interaction, as the deletion of T6SS-2 genes led to a much higher level of organ infection. It was demonstrated that the T6SS-2 was not involved in human intestinal epithelial cell invasion and intracellular survival in macrophages. Therefore, additional mechanisms of T6SS-2 used to interact with host interaction need to be investigated in the future. Since the T6SS-2 gene cluster contains 4 pilus-associated genes, these four flagellin genes (fimA4, fimB, fimD, and fimA5) expressions were compared with wild-type and T6SS-2-deficient mutant (Δ*vasK*2). The quantitative real-time PCR result showed that the expression level of pilus was decreased in mutant, suggesting that the expressions of the T6SS-2 and these four pilus genes were coordinated (Supplementary Figure S4). FimA is a potent inducer of pro-inflammatory cytokines involved in tissue destruction (Choi et al., 2016). In T6SS-2 of *C. sakazakii* ATCC 12868, two of the four pili genes encode the FimA protein. We propose that low expression levels of FimA protein in T6SS-2-deficient mutant strains may help bacterium evade the host immune response which result in the high pathogenicity (Viscount et al., 1997). The exact molecular mechanism will be explored in the future.

## Author Contributions

BL and MW conceived and designed the experiments. MW and HC performed the experiments and analyzed the data. QW, TX, and XG prepared the strain samples. MW, HC, and BL prepared the manuscript. All authors read and approved the final manuscript.

## Conflict of Interest Statement

The authors declare that the research was conducted in the absence of any commercial or financial relationships that could be construed as a potential conflict of interest.

## Footnotes

1 http://db-mml.sjtu.edu.cn/SecReT6/
