# Supplementary material for: The Roles of Two Type VI Secretion Systems in Cronobacter sakazakii ATCC 12868
Source: Front Microbiol. 2018 Oct 22;9:2499. doi: 10.3389/fmicb.2018.02499 (PMC6204376; doi:10.3389/fmicb.2018.02499)
Supplement: Supplementary file 1 [file Table_1.docx]

The Roles of Two Type VI Secretion Systems in *Cronobacter sakazakii* ATCC 12868

Min Wang^1,2,3†^, Hengchun Cao^1,2,3†^, Qian Wang^1,2,3^, Tingting Xu^1,2,3^, Xi Guo^1,2,3^, Bin Liu^1,2,3^*

^1^Key Laboratory of Molecular Microbiology and Technology, Ministry of Education, Tianjin Economic-Technological Development Area, Tianjin, China

^2^Tianjin Key Laboratory of Microbial Functional Genomics, Tianjin Economic-Technological Development Area, Tianjin, China

^3^TEDA Institute of Biological Sciences and Biotechnology, Nankai University, Tianjin Economic-Technological Development Area, Tianjin, China

^†^These authors contributed equally to this work.

*Correspondence: liubin1981@nankai.edu.cn

Number of supplementary figures/tables: 7

**Supplementary Figure S1: Growth curve of WT and T6SS-deficient strains.** It was shown that there are no differences between the wild type and the two T6SS-deficient strains.

**Supplementary Figure S2: *C. sakazakii* is highly virulent to *E. coli* O157:H7 in a T6SS-dependent manner.** Survival of streptomycin-resistant *E. coli* was determined by measuring CFU following exposure to the predator listed on the *x* axis.

**Supplementary Figure S3: Restoration of T6SS-2 genes (*vasK2* and *hcp*) in trans complements the survival defect of mutant strains.** The result showed T6SS-2 is involved in bacterial colonization in tissues of neonatal rats. Asterisks indicate statistically significant differences between the mutant, wild type and complement as determined by one-way ANOVA with Dunnett’s posttest.

**Supplementary Figure S4: Expression of four pili-related genes in WT and T6SS-deficient strains.** The result showed that the expression level was decreased in T6SS-2-deficient mutant.

**Supplementary Table S1: Bacterial strains and plasmids used in this study**

| **Species Name** | | **Strain** | | **Source** | | **GenBank assembly accession** |
| --- | --- | --- | --- | --- | --- | --- |
| **138 *C. sakazakii* strains used for analysis of T6SS distribution** | | | | | | |
| *C. sakazakii* | ATCC BAA-894 | | PIF | | | GCA_000017665.1 |
| *C. sakazakii* | E899 | | clinical | | | GCA_000214745.2 |
| *C. sakazakii* | ES15 | | whole grains | | | GCA_000263215.1 |
| *C. sakazakii* | 680 | | clinical | | | GCA_000319615.1 |
| *C. sakazakii* | Sp291 | | PIF manufacturing enviroment | | | GCA_000339015.1 |
| *C. sakazakii* | E764 | | clinical | | | GCA_000409245.1 |
| *C. sakazakii* | 2151 | | clinical(cerebrospinal ﬂuid) | | | GCA_000409265.1 |
| *C. sakazakii* | ES713 | | PIF | | | GCA_000409385.1 |
| *C. sakazakii* | ES35 | | clinical | | | GCA_000409405.1 |
| *C. sakazakii* | NCIMB 8272 | | milk powder | | | GCA_000463095.2 |
| *C. sakazakii* | 8399 | | cerebral spinal ﬂuid | | | GCA_000467775.2 |
| *C. sakazakii* | NBRC 102416 | | unknown | | | GCA_000684935.1 |
| *C. sakazakii* | HPB5174 | | Powdered Infant Formula Facility | | | GCA_000698225.1 |
| *C. sakazakii* | NM1240 | | Meningitis infant | | | GCA_000974965.1 |
| *C. sakazakii* | NCTC 8155 | | unknown | | | GCA_001277275.1 |
| *C. sakazakii* | 702 | | stools | | | GCA_001308935.1 |
| *C. sakazakii* | 712 | | prepared formula | | | GCA_001308945.1 |
| *C. sakazakii* | 767 | | Trachea | | | GCA_001308955.1 |
| *C. sakazakii* | 707 | | skin | | | GCA_001308965.1 |
| *C. sakazakii* | 695 | | Trachea | | | GCA_001309015.1 |
| *C. sakazakii* | 709 | | Trachea | | | GCA_001309035.1 |
| *C. sakazakii* | 706 | | stools | | | GCA_001309045.1 |
| *C. sakazakii* | 705 | | Trachea | | | GCA_001309055.1 |
| *C. sakazakii* | 699 | | Trachea | | | GCA_001309095.1 |
| *C. sakazakii* | 700 | | stools | | | GCA_001309115.1 |
| *C. sakazakii* | 694 | | conjunctivae | | | GCA_001309135.1 |
| *C. sakazakii* | 690 | | stools | | | GCA_001309155.1 |
| *C. sakazakii* | 693 | | stools | | | GCA_001309175.1 |
| *C. sakazakii* | 715 | | prepared formula | | | GCA_001309185.1 |
| *C. sakazakii* | 691 | | sputum | | | GCA_001309215.1 |
| *C. sakazakii* | 698 | | stools | | | GCA_001309225.1 |
| *C. sakazakii* | 716 | | Infant formula | | | GCA_001309235.1 |
| *C. sakazakii* | 730 | | stools | | | GCA_001309275.1 |
| *C. sakazakii* | 692 | | stools | | | GCA_001309295.1 |
| *C. sakazakii* | 714 | | end of bottle | | | GCA_001309305.1 |
| *C. sakazakii* | 696 | | stools | | | GCA_001309315.1 |
| *C. sakazakii* | 713 | | end of bottle | | | GCA_001309345.1 |
| *C. sakazakii* | 703 | | Trachea | | | GCA_001309375.1 |
| *C. sakazakii* | 708 | | Trachea | | | GCA_001309385.1 |
| *C. sakazakii* | 711 | | stools | | | GCA_001309415.1 |
| *C. sakazakii* | 701 | | Peritoneal fluid | | | GCA_001309435.1 |
| *C. sakazakii* | wls-N2695 | | unknown | | | unpublished data |
| *C. sakazakii* | wls-N2699 | | unknown | | | unpublished data |
| *C. sakazakii* | wls-N2693 | | unknown | | | unpublished data |
| *C. sakazakii* | wls-N2694 | | unknown | | | unpublished data |
| *C. sakazakii* | G2356 | | unknown | | | unpublished data |
| *C. sakazakii* | G2538 | | skimmed milk power | | | unpublished data |
| *C. sakazakii* | G2539 | | Throat | | | unpublished data |
| *C. sakazakii* | G2591 | | milk powder | | | unpublished data |
| *C. sakazakii* | G2593 | | whey power | | | unpublished data |
| *C. sakazakii* | G2594 | | milk power | | | unpublished data |
| *C. sakazakii* | G2595 | | unknown | | | unpublished data |
| *C. sakazakii* | G2726 | | skimmed milk power | | | unpublished data |
| *C. sakazakii* | G2727 | | skimmed milk power | | | unpublished data |
| *C. sakazakii* | G2729 | | milk power | | | unpublished data |
| *C. sakazakii* | G2730 | | whey power | | | unpublished data |
| *C. sakazakii* | G2731 | | foot wound | | | unpublished data |
| *C. sakazakii* | G2733 | | Bronchial secretion | | | unpublished data |
| *C. sakazakii* | G3862 | | shrimp strips | | | unpublished data |
| *C. sakazakii* | G3863 | | milk power | | | unpublished data |
| *C. sakazakii* | G3865 | | tea | | | unpublished data |
| *C. sakazakii* | G3866 | | milk power | | | unpublished data |
| *C. sakazakii* | G3867 | | milk power | | | unpublished data |
| *C. sakazakii* | G3868 | | soy bean | | | unpublished data |
| *C. sakazakii* | G3869 | | onion rings | | | unpublished data |
| *C. sakazakii* | G3870 | | chocolate | | | unpublished data |
| *C. sakazakii* | G3878 | | milk power | | | unpublished data |
| *C. sakazakii* | G3879 | | unknown | | | unpublished data |
| *C. sakazakii* | G3880 | | milk power | | | unpublished data |
| *C. sakazakii* | G3881 | | milk power | | | unpublished data |
| *C. sakazakii* | G3883 | | chocolate | | | unpublished data |
| *C. sakazakii* | G3885 | | unknown | | | unpublished data |
| *C. sakazakii* | G3888 | | unknown | | | unpublished data |
| *C. sakazakii* | G3942 | | milk power | | | unpublished data |
| *C. sakazakii* | G3943 | | milk power | | | unpublished data |
| *C. sakazakii* | G3945 | | milk power | | | unpublished data |
| *C. sakazakii* | G3946 | | milk power | | | unpublished data |
| *C. sakazakii* | G3976 | | shrimp strips | | | unpublished data |
| *C. sakazakii* | G3978 | | milk power | | | unpublished data |
| *C. sakazakii* | G3980 | | unknown | | | unpublished data |
| *C. sakazakii* | G3982 | | milk power | | | unpublished data |
| *C. sakazakii* | G3984 | | milk power | | | unpublished data |
| *C. sakazakii* | G3987 | | milk powder | | | unpublished data |
| *C. sakazakii* | G3988 | | milk power | | | unpublished data |
| *C. sakazakii* | G3989 | | milk power | | | unpublished data |
| *C. sakazakii* | G3992 | | milk power | | | unpublished data |
| *C. sakazakii* | G4023 | | cheese | | | unpublished data |
| *C. sakazakii* | G4034 | | cookies | | | unpublished data |
| *C. sakazakii* | G4035 | | cookies | | | unpublished data |
| *C. sakazakii* | G4038 | | butter | | | unpublished data |
| *C. sakazakii* | G4039 | | butter | | | unpublished data |
| *C. sakazakii* | G4041 | | milk power | | | unpublished data |
| *C. sakazakii* | G4042 | | unknown | | | unpublished data |
| *C. sakazakii* | G4043 | | cookies | | | unpublished data |
| *C. sakazakii* | G4045 | | cookies | | | unpublished data |
| *C. sakazakii* | G4047 | | cheese | | | unpublished data |
| *C. sakazakii* | G4048 | | cheese | | | unpublished data |
| *C. sakazakii* | G4050 | | cheese | | | unpublished data |
| *C. sakazakii* | G4051 | | cheese | | | unpublished data |
| *C. sakazakii* | G4053 | | cheese | | | unpublished data |
| *C. sakazakii* | G4054 | | cheese | | | unpublished data |
| *C. sakazakii* | G4056 | | cheese | | | unpublished data |
| *C. sakazakii* | G4057 | | cheese | | | unpublished data |
| *C. sakazakii* | G4058 | | flavorings | | | unpublished data |
| *C. sakazakii* | G4059 | | cheese | | | unpublished data |
| *C. sakazakii* | G4060 | | cheese | | | unpublished data |
| *C. sakazakii* | G4063 | | ice cream | | | unpublished data |
| *C. sakazakii* | G4064 | | ice cream | | | unpublished data |
| *C. sakazakii* | G4065 | | cookies | | | unpublished data |
| *C. sakazakii* | G4066 | | cheese | | | unpublished data |
| *C. sakazakii* | G4067 | | cheese | | | unpublished data |
| *C. sakazakii* | G4068 | | milk power | | | unpublished data |
| *C. sakazakii* | G4078 | | milk power | | | unpublished data |
| *C. sakazakii* | G4079 | | milk power | | | unpublished data |
| *C. sakazakii* | G4081 | | milk power | | | unpublished data |
| *C. sakazakii* | G4082 | | whey power | | | unpublished data |
| *C. sakazakii* | G4084 | | whey power | | | unpublished data |
| *C. sakazakii* | G4088 | | milk power | | | unpublished data |
| *C. sakazakii* | G4089 | | milk power | | | unpublished data |
| *C. sakazakii* | G4021 | | ice cream | | | unpublished data |
| *C. sakazakii* | G4026 | | cheese | | | unpublished data |
| *C. sakazakii* | G3990 | | milk powder | | | unpublished data |
| *C. sakazakii* | G2375 | | instant noodle | | | unpublished data |
| *C. sakazakii* | G4024 | | cookies | | | unpublished data |
| *C. sakazakii* | G4036 | | cookies | | | unpublished data |
| *C. sakazakii* | G4033 | | cookies | | | unpublished data |
| *C. sakazakii* | G4032 | | flavirings | | | unpublished data |
| *C. sakazakii* | G4037 | | cookies | | | unpublished data |
| *C. sakazakii* | G4062 | | milk powder | | | unpublished data |
| *C. sakazakii* | G4085 | | milk powder | | | unpublished data |
| *C. sakazakii* | G4091 | | skimmed milk powder | | | unpublished data |
| *C. sakazakii* | G2705 | | milk powder | | | unpublished data |
| *C. sakazakii* | G2706 | | milk powder | | | unpublished data |
| *C. sakazakii* | G2708 | | milk powder | | | unpublished data |
| *C. sakazakii* | G3884 | | milk powder | | | unpublished data |
| *C. sakazakii* | G4030 | | chocolate | | | unpublished data |
| *C. sakazakii* | G4031 | | milk powder | | | unpublished data |
| *C. sakazakii* | G4027 | | cheese | | | unpublished data |
| **plasmids** | | | | | | |
| pKD3 | Containing a chloramphenicol resistance cassette and the flipase recognition sites, CmR | | | | | |
| pKD46 | Red recombination plasmid, ApR | | | | | |
| pTrc99A | Expression vector, ApR | | | | | |
| pLW4371 | pTrc99A carrying *vask1*, ApR | | | | | |
| pLW4372 | pTrc99A carrying *hcp1*, ApR | | | | | |
| pLW4373 | pTrc99A carrying *vask2*, ApR | | | | | |
| pLW4374 | pTrc99A carrying *hcp2*, ApR | | | | | |
| **Strains constructed in this study** | | | | | | |
| *C. sakazakii* | H4821 | | this lab | | *vasK*1 deletion mutant in G2595 catR | |
| *C. sakazakii* | H4822 | | this lab | | *hcp*1 deletion mutant in G2595 catR | |
| *C. sakazakii* | H4823 | | this lab | | *vasK*2 deletion mutant in G2595 catR | |
| *C. sakazakii* | H4824 | | this lab | | *hcp*2 deletion mutant in G2595 catR | |
| *C. sakazakii* | H6561 | | this lab | | H4821 contain plasmid pLW4371 | |
| *C. sakazakii* | H6562 | | this lab | | H4821 contain plasmid pTrc99A | |
| *C. sakazakii* | H6563 | | this lab | | H4822 contain plasmid pLW4372 | |
| *C. sakazakii* | H6564 | | this lab | | H4822 contain plasmid pTrc99A | |
| *C. sakazakii* | H6565 | | this lab | | H4823 contain plasmid pLW4373 | |
| *C. sakazakii* | H6566 | | this lab | | H4823 contain plasmid pTrc99A | |
| *C. sakazakii* | H6567 | | this lab | | H4824 contain plasmid pLW4374 | |
| *C. sakazakii* | H6568 | | this lab | | H4824 contain plasmid pTrc99A | |

**Supplementary Table S2: Primers used in this study**

| **Genes F/R sequences** |
| --- |
| **Primers for gene mutation**  *vasK*1 F taaagcgctgcaaaccaccgccgggcgcgatctcttcagcgtgtaggctggagctgcttc  *vasK*1 R gctaatcgcagggtaatgactcattatccgttccttgcggcatatgaatatcctccttag  *hcp*1 F ttcgaaagcaataatcgtcattaacgaagagtaaatattgtgtaggctggagctgcttc  *hcp*1 R cctgaaagggtaaagtgagcccttgcgggctcactacgacatatgaatatcctccttag  *vasK*2 F gaacgggaactatcgtccttgcaaacatactcatcccccggtgtaggctggagctgcttc  *vasK*2 R tcacgatgcgcagggacgttcacaaaaccgacgcgttgacatatgaatatcctccttag  *hcp*2 F gccgtcagataacggcaagttttggacttcaggcagttagtgtaggctggagctgcttc  *hcp*2 R atcgcttgttttaaggtggcgttattcgccatttatagtcatatgaatatcctccttag  **Primers for identification of the gene mutations**  *vasK*1 F1 acgtgattttccaggaagcgg  *vasK*1 R1 ggagtgaataccacgacgat  *vasK*1 F2 atactgacgccctacgctgtc  *vasK*1 R2 attggctgagacgaaaaaca  *hcp*1 F1 aaacatgctgtcaggctgagag  *hcp*1 R1 ggagtgaataccacgacgat  *hcp*1 F2 cagcgcagcgtggaaagaaac  *hcp*1 R2 attggctgagacgaaaaaca  *vasK*2 F1 ggaagagcacacgtctgaact  *vasK*2 R1 ggagtgaataccacgacgat  *vasK*2 F2 ttccggtgaaagtatcgctgg  *vasK*2 R2 attggctgagacgaaaaaca  *hcp*2 F1 atactgtgtaattgcgcgtccg  *hcp*2 R1 ggagtgaataccacgacgat  *hcp*2 F2 tggaaggattaacggtgtcgt  *hcp*2 R2 attggctgagacgaaaaaca  **Primers for gene cloning**  *vasK1* F catgccatggatgctgaatattctcctttc  *vasK1* R gctctagattaagggcatgcgaagccag  *hcp1* F catgccatggatggctattgatatgttcct  *hcp1* R gctctagattacgcttctttgttttctt  *vasK2* F catgccatggatggcttttttgaatcggtta  *vasK2* R gctctagattatgccgccgccgggcagg  *hcp2* F catgccatggatggcacaggatatgtttatt  *hcp2* R gctctagattagccgtgcaggttagcttt |

**Supplementary Table S3: T6SS-1 and T6SS-2 gene annotations**

T6SS-1 genes

| Gene  name | Location | Strand | Similar protein (strains) | Genbank accession number | %Identical/  %Similar | Putative functions |
| --- | --- | --- | --- | --- | --- | --- |
| tssJ | 4129538..4130044 | + | type VI secretion system-associated lipoprotein [Cronobacter ATCC BAA-894] | ABU79134.1 | 100.00/100.00 | type VI secretion system-associated lipoprotein |
| tssK | 4130069..4131412 | + | type VI secretion system-associated protein [Cronobacter sakazakii ATCC BAA-894] | ABU79133.1 | 100.00/100.00 | type VI secretion system-associated protein |
| tssL | 4131430..4132665 | + | ImpJ [Cronobacter sakazakii ES15] | CCK04631.1 | 100.00/100.00 | Outer membrane protein |
| vasK | 4132667..4136287 | + | type VI secretion system membrane subunit | WP_069683178.1 | 100.00/100.00 | type VI secretion system membrane subunit |
| tagF | 4136304..4137014 | + | type VI secretion system-associated protein TagF [Cronobacter] | WP_004385967.1 | 100.00/100.00 | type VI secretion system-associated protein TagF |
| tssA | 4137024..4138046 | + | type VI secretion protein ImpA [Cronobacter sakazakii] | CCK10526.1 | 100.00/100.00 | type VI secretion protein ImpA |
| tssB | 4138110..4138646 | + | hypothetical protein ESA_03942 [Cronobacter sakazakii ATCC BAA-894] | ABU79128.1 | 100.00/100.00 | type VI secretion system contractile sheath small subunit |
| tssC | 4138650..4140149 | + | hypothetical protein ESA_03941 [Cronobacter sakazakii ATCC BAA-894] | ABU79127.1 | 100.00/100.00 | type VI secretion system contractile sheath large subunit |
| orf1 | 4140628..4140897 | + | hypothetical protein [Cronobacter] | WP_032970327.1 | 100.00/100.00 | hypothetical protein |
| tae4 | 4140894..4141388 | + | hypothetical protein [Cronobacter] | WP_032970324.1 | 100.00/100.00 | Type VI secretion system (T6SS), amidase effector protein 4 |
| hcp | 4141646..4142128 | + | hypothetical protein ESA_03934 [Cronobacter sakazakii ATCC BAA-894] | ABU79120.1  WP_004386969.1 | 100.00/100.00 | Hcp1 family type VI secretion system effector |
| tai4 | 4142176..4142640 | + | hypothetical protein [Cronobacter sakazakii] | WP_069683181.1 | 100.00/99.20 | Type VI secretion system (T6SS), amidase immunity protein |
| tagH | 4142898..4144814 | + | type VI secretion system-associated FHA domain protein TagH [Cronobacter sakazakii] | WP_069683179.1 | 100.00/100.00 | type VI secretion system-associated FHA domain protein TagH |
| ptc1 | 4144811..4145605 | + | serine/threonine phosphatase [Cronobacter] | WP_004386964.1 | 100.00/100.00 | serine/threonine phosphatase |
| Orf2 | 4145624..4146628 | + | hypothetical protein [Cronobacter sakazakii] | WP_069683180.1 | 100.00/100.00 | hypothetical protein |
| tagJ | 4146654..4147487 | + | hypothetical protein [Cronobacter] | WP_023897738.1 | 100.00/100.00 | ImpE protein |
| tssE | 4147480..4148052 | + | type VI secretion system protein ImpF [Cronobacter] | WP_004386961.1 | 100.00/100.00 | type VI secretion system protein ImpF |
| tssF | 4148055..4149926 | + | type VI secretion system protein ImpG [Cronobacter] | WP_004386960.1 | 100.00/100.00 | type VI secretion system protein ImpG |
| tssG | 4149923..4150966 | + | type VI secretion system baseplate subunit TssG [Cronobacter] | WP_004386959.1 | 100.00/100.00 | type VI secretion system baseplate subunit TssG |
| Orf3 | 4151039..4151356 | + | hypothetical protein [Cronobacter] | WP_007872403.1 | 100.00/100.00 | hypothetical protein |
| tssH | 4151403..4154018 | + | type VI secretion system ATPase TssH [Cronobacter sakazakii] | WP_069683978.1 | 100.00/100.00 | type VI secretion system ATPase TssH |

T6SS-2 gene clusters

| Gene  name | Location | Strand | Similar protein (strains) | Genbank accession number | %Identical/  %Similar | Putative functions |
| --- | --- | --- | --- | --- | --- | --- |
| tssE | 651636..652118 | - | hypothetical protein [Cronobacter sakazakii] | WP_007848162.1 | 100.00/100.00 | type VI secretion system baseplate subunit TssE |
| tagJ | 652141..652933 | - | hypothetical protein ESA_02736 [Cronobacter sakazakii ATCC BAA-894] | ABU77968.1 | 100.00/100.00 | ImpE family T6SS protein Cts1E |
| tagK | 652998..653786 | - | TagK domain-containing protein [Cronobacter sakazakii] | WP_069684078.1 | 100.00/100.00 | TagK domain-containing protein |
| ppaR | 653792..654052 | - | hypothetical protein [Cronobacter sakazakii] | WP_004385553.1 | 100.00/100.00 | PAAR motif protein |
| tssI | 654110..656612 | - | hypothetical protein BN133_4141 [Cronobacter dublinensis 582] | CCJ87764.1 | 86.80/79.40 | hypothetical protein |
| Orf1 | 656652..657312 | - |  |  |  |  |
| vasK | 657371..661105 | - | type VI secretion system membrane subunit [Cronobacter sakazakii] | WP_085047032.1 | 99.90/99.90 | type VI secretion system membrane subunit |
| tssL | 661168..662502 | - | type VI secretion system protein TssL [Cronobacter sakazakii] | WP_063264942.1 | 100.00/100.00 | type VI secretion system protein TssL |
| tssK | 662511..663857 | - | type VI secretion system baseplate subunit TssK [Cronobacter sakazakii] | WP_007848145.1 | 100.00/100.00 | type VI secretion system baseplate subunit TssK |
| tssJ | 663844..664329 | - | type VI secretion system lipoprotein TssJ [Cronobacter sakazakii] | WP_097649158.1 | 100.00/100.00 | type VI secretion system lipoprotein TssJ |
| Orf1 | 664419..664535 | - | hypothetical protein BN128_4257 [Cronobacter sakazakii 696] | CCK05943.1 | 100.00/100.00 | hypothetical protein |
| hcp | 664557..665042 | - | Hcp1 family type VI secretion system effector [Cronobacter] | WP_007791657.1 | 100.00/100.00 | Hcp1 family type VI secretion system effector |
| tssC | 665201..666703 | - | type VI secretion system contractile sheath large subunit [Cronobacter sakazakii] | WP_069682551.1 | 100.00/99.80 | type VI secretion system contractile sheath large subunit |
| tssB | 666720..667250 | - | type VI secretion system contractile sheath small subunit [Cronobacter sakazakii] | WP_069682569.1 | 100.00/100.00 | type VI secretion system contractile sheath small subunit |
| fimA5 | 667340..667804 | - | type 1 fimbrial protein [Cronobacter sakazakii] | WP_038876730.1 | 100.00/99.40 | type 1 fimbrial protein |
| fimD | 667921..670521 | - | fimbrial biogenesis outer membrane usher protein [Cronobacter sakazakii] | WP_069682549.1 | 100.00/100.00 | fimbrial biogenesis outer membrane usher protein |
| fimB | 670609..671079 | - | pilus assembly protein , partial [Cronobacter sakazakii] | WP_076738072.1 | 100.00/100.00 | pilus assembly protein |
| orf3 | 671240..671380 | + | hypothetical protein BN128_4250 [Cronobacter sakazakii 696] | CCK05936.1 | 100.00/97.80 | hypothetical protein |
| fimA4 | 671454..671984 | - | type 1 fimbrial protein [Cronobacter sakazakii] | WP_007865470.1 | 100.00/100.00 | type 1 fimbrial protein |
| tssH | 672048..674804 | - | type VI secretion system ATPase TssH [Cronobacter sakazakii] | WP_069682548.1 | 99.20/98.90 | type VI secretion system ATPase TssH |
| tssF | 675272..677152 | + | type VI secretion system baseplate subunit TssF [Cronobacter sakazakii] | WP_069682547.1 | 100.00/100.00 | type VI secretion system baseplate subunit TssF |
| tssG | 677152..678180 | + | type VI secretion system baseplate subunit TssG [Cronobacter sakazakii] | WP_105607601.1 | 99.70/99.70 | type VI secretion system baseplate subunit TssG [Cronobacter sakazakii] |
| tssA | 678280..679281 | + | hypothetical protein [Cronobacter sakazakii] | WP_069682545.1 | 100.00/100.00 | ImpA, N-terminal, type VI secretion system |
