# Supplementary figures and images for: The Roles of Two Type VI Secretion Systems in Cronobacter sakazakii ATCC 12868
Source: Front Microbiol. 2018 Oct 22;9:2499. doi: 10.3389/fmicb.2018.02499 (PMC6204376; doi:10.3389/fmicb.2018.02499)

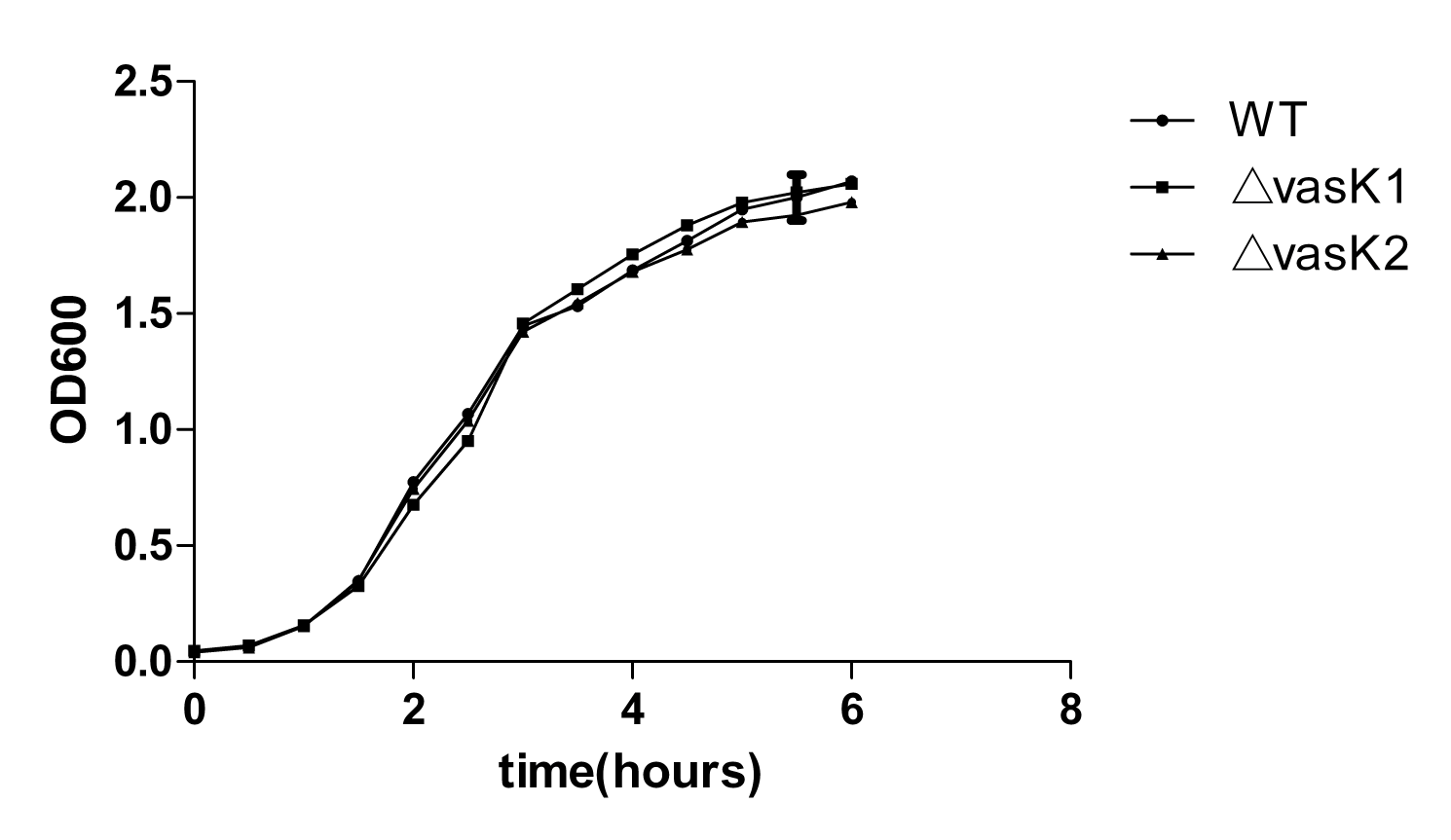

Supplement: Supplementary file 2 [file Image_1.TIF]

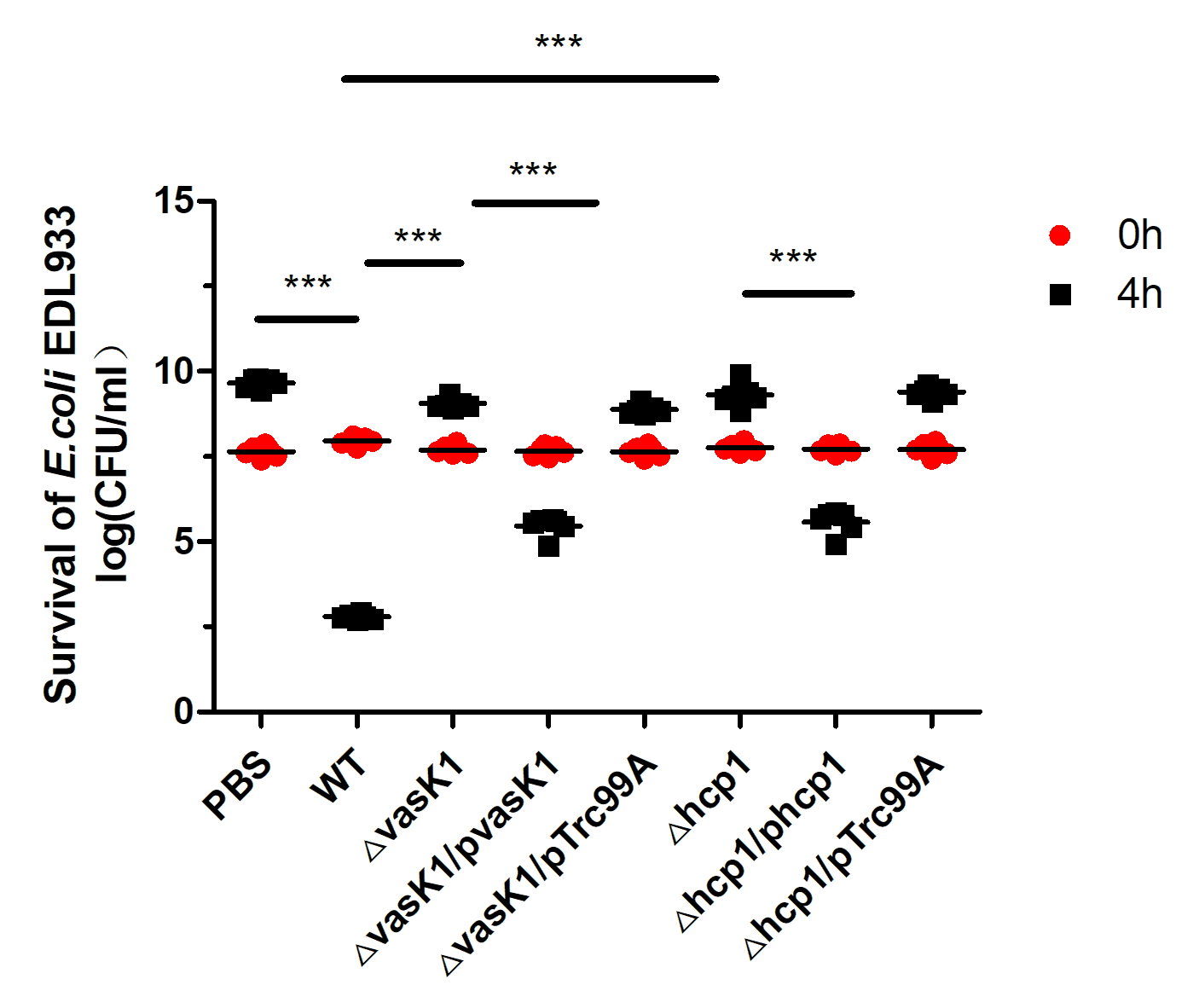

Supplement: Supplementary file 3 [file Image_2.TIF]

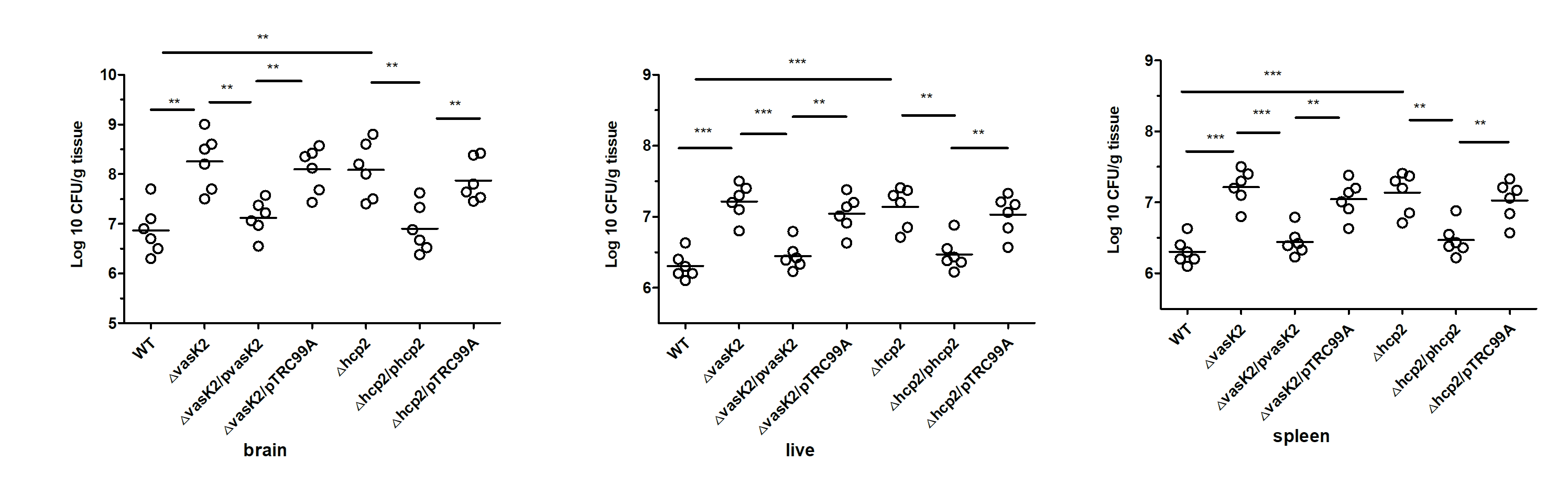

Supplement: Supplementary file 4 [file Image_3.TIF]

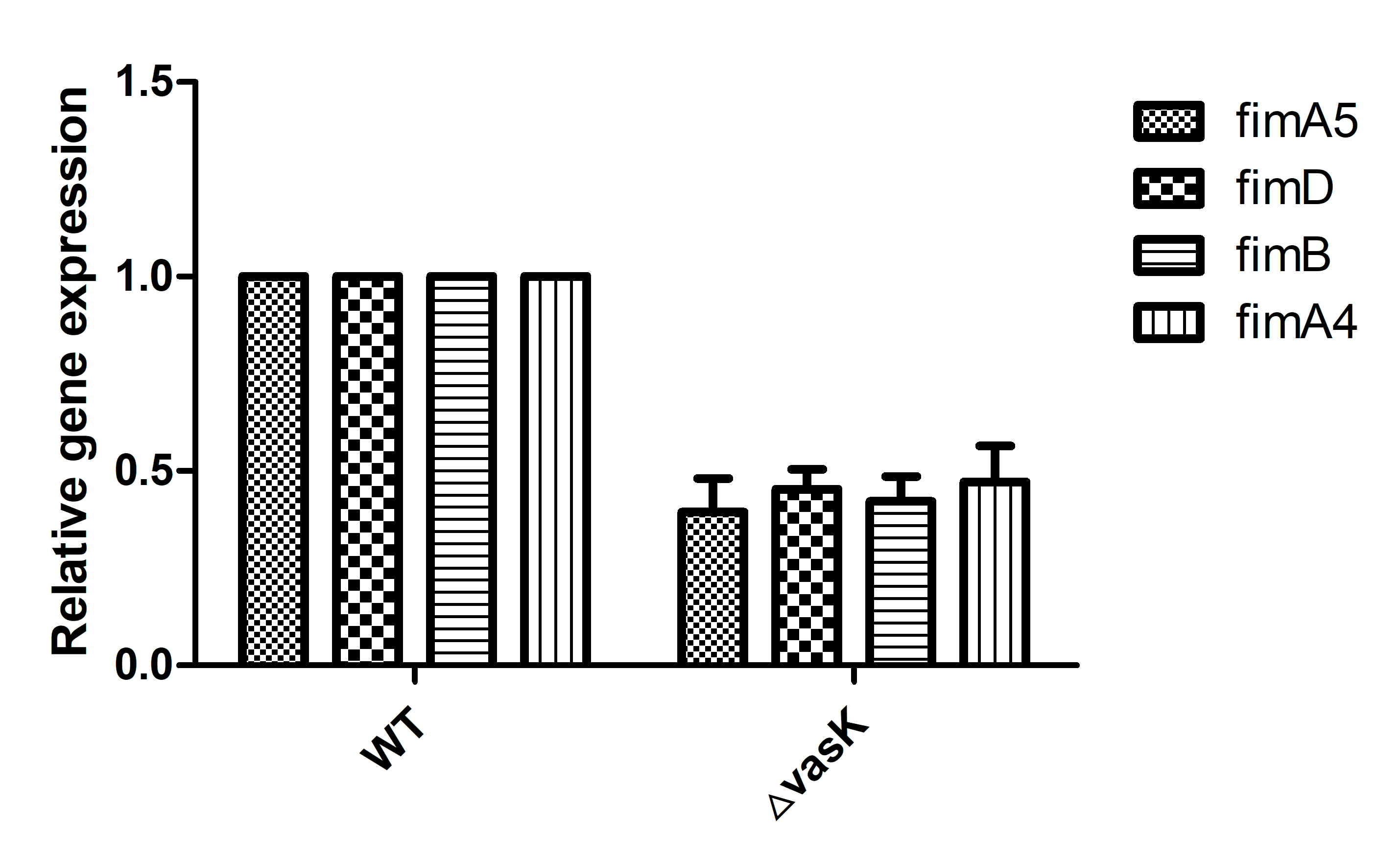

Supplement: Supplementary file 5 [file Image_4.TIF]
